# Knowledge, Attitude and Practice Toward COVID-19 Among the Public in the Kingdom of Saudi Arabia: A Cross-Sectional Study

**DOI:** 10.3389/fpubh.2020.00217

**Published:** 2020-05-27

**Authors:** Mohammed K. Al-Hanawi, Khadijah Angawi, Noor Alshareef, Ameerah M. N. Qattan, Hoda Z. Helmy, Yasmin Abudawood, Mohammed Alqurashi, Waleed M. Kattan, Nasser Akeil Kadasah, Gowokani Chijere Chirwa, Omar Alsharqi

**Affiliations:** ^1^Department of Health Services and Hospital Administration, Faculty of Economics and Administration, King Abdulaziz University, Jeddah, Saudi Arabia; ^2^Department of Business Administration, Faculty of Economics and Administration, King Abdulaziz University, Jeddah, Saudi Arabia; ^3^Centre for Health Economics, University of York, Heslington, United Kingdom; ^4^Economics Department, Chancellor College, University of Malawi, Zomba, Malawi

**Keywords:** COVID-19, KAP, Saudi Arabia, public adherence, health education intervention, pandemic reaction, preventive measures

## Abstract

**Background:** Saudi Arabia has taken unprecedented and stringent preventive and precautionary measures against COVID-19 to control its spread, safeguard citizens and ensure their well-being. Public adherence to preventive measures is influenced by their knowledge and attitude toward COVID-19. This study investigated the knowledge, attitudes, and practices of the Saudi public, toward COVID-19, during the pandemic.

**Methods:** This is a cross-sectional study, using data collected via an online self-reported questionnaire, from 3,388 participants. To assess the differences in mean scores, and identify factors associated with knowledge, attitudes, and practices toward COVID-19, the data were run through univariate and multivariable regression analyses, respectively.

**Results:** The majority of the study participants were knowledgeable about COVID-19. The mean COVID-19 knowledge score was 17.96 (SD = 2.24, range: 3–22), indicating a high level of knowledge. The mean score for attitude was 28.23 (SD = 2.76, range: 6–30), indicating optimistic attitudes. The mean score for practices was 4.34 (SD = 0.87, range: 0–5), indicating good practices. However, the results showed that men have less knowledge, less optimistic attitudes, and less good practice toward COVID-19, than women. We also found that older adults are likely to have better knowledge and practices, than younger people.

**Conclusions:** Our finding suggests that targeted health education interventions should be directed to this particular vulnerable population, who may be at increased risk of contracting COVID-19. For example, COVID-19 knowledge may increase significantly if health education programs are specifically targeted at men.

## Background

Coronavirus disease 2019 (COVID-19) is defined as an illness caused by a novel coronavirus, now called Severe Acute Respiratory Syndrome Coronavirus 2 (SARS-CoV-2; formerly called 2019-nCoV). COVID-19 is an emerging respiratory infection that was first discovered in December 2019, in Wuhan city, Hubei Province, China (1). SARS-CoV-2 belongs to the larger family of ribonucleic acid (RNA) viruses, leading to infections, from the common cold, to more serious diseases, such as Middle East Respiratory Syndrome (MERS-CoV) and Severe Acute Respiratory Syndrome (SARS-CoV) (2). The main symptoms of COVID-19 have been identified as fever, dry cough, fatigue, myalgia, shortness of breath, and dyspnoea (3, 4).

COVID-19 is characterized by rapid transmission, and can occur by close contact with an infected person (5–9). The details on the disease are evolving. As such, this may not be the only way the transmission is occurring. COVID-19 has spread widely and rapidly, from Wuhan city, to other parts of the world, threatening the lives of many people (10). By the end of January 2020, the World Health Organization (WHO) announced a public health emergency of international concern and called for the collaborative effort of all countries, to prevent its rapid spread. Later, the WHO declared COVID-19 a “global pandemic” (11).

Following the WHO declaration, countries around the globe, including the Kingdom of Saudi Arabia (KSA), have been leaning on response plans to respond to the pandemic and contain the virus. Following the confirmation of its first case of COVID-19, on Monday 2 March 2020, the Saudi government has been vigilantly monitoring the situation and developing country-specific measures that are in line with the WHO guidelines in dealing with the outbreak (12). These includes suspending all inbounds and outbounds flights, closing all malls and shops in the country, except pharmacies and grocery stores, and closing down schools and universities. Umrah visas have been suspended, as have prayers at mosques, including the two Holy Mosques in Mekkah and Almadina. On 24 March 2020, the government imposed a nationwide curfew to restrict people movements for most of the day hours.

Despite the unprecedented national measures in combating the outbreak, the success or failure of these efforts is largely dependent on public behavior. Specifically, public adherence to preventive measures established by the government is of prime importance to prevent the spread of the disease. Adherence is likely to be influenced by the public's knowledge and attitudes toward COVID-19. Evidence shows that public knowledge is important in tackling pandemics (13, 14). By assessing public awareness and knowledge about the coronavirus, deeper insights into existing public perception and practices can be gained, thereby helping to identify attributes that influence the public in adopting healthy practices and responsive behavior (15). Assessing public knowledge is also important in identifying gaps and strengthening ongoing prevention efforts. Thus, this study aims to investigate the knowledge, attitudes and practices (KAP) of KSA residents, toward COVID-19 during the pandemic spike.

To the researchers' knowledge, this is the first study to investigate COVID-19 KAP, and associated sociodemographic characteristics among the general population of the KSA. The findings of this study are expected to provide useful information to policymakers, about KAP among the Saudi population, at this critical time. The findings may also inform public health officials on further public health interventions, awareness, and policy improvements pertaining to the COVID-19 outbreak.

## Materials and Methods

### Study Design and Sample

This cross-sectional study was conducted among the general population of Saudi Arabia, from 20 March 2020, to 24 March 2020. Given the social distancing (physical distancing) measures and restricted movement and lockdowns, data were collected online, via a self-reported questionnaire, using SurveyMonkey. Given the high internet usage among people in the KSA, a link to the survey was distributed to respondents, via Twitter and WhatsApp groups. The link was also posted on the King Abdulaziz University website.

The larger the target sample size, the higher the external validity and the greater the generalizability of the study (16). This study aimed to maximize reach and gather data from as many respondents as possible. According to the latest KSA census, Saudi Arabia has a population of 34,218,169 (17). The representative target sample size needed, to achieve the study objectives and sufficient statistical power, was calculated with a sample size calculator (18). The sample size calculator arrived at 1,037 participants, using a margin of error of ±4%, a confidence level of 99%, a 50% response distribution, and 34,218,169 people.

### Measurement Tool and Data Analysis

The self-reported questionnaire was developed by the authors, according to guidelines for the community of COVID-19, by the Centers for Disease Control and Prevention (CDC) (19). The questionnaire was conducted in Arabic language. It was initially drafted in English by H.Z.H., and Y.A., and was translated from English to Arabic by M.K.A and M.A. The questionnaire was translated then back to English by N.A and W.K to ensure the meaning of the content.

On the first page of the online questionnaire, respondents were clearly informed about the background and objectives of the study. Respondents were informed that they were free to withdraw at any time, without giving a reason, and that all information and opinions provided would be anonymous and confidential. Respondents living in Saudi Arabia, aged 18 years or older, understand the content of the questionnaire, and agree to participate in the study were instructed to complete the questionnaire. Online informed consent were obtained before proceeding with the questionnaire.

The questionnaire consisted of four primary sections. The first section gathered information on respondents' sociodemographic characteristics, including age, gender, marital status, education level, work status, region of residence, and income level. The second section assessed participants' knowledge of COVID-19. This section included 22 items on modes of transmission, clinical symptoms, treatment, risk groups, isolation, prevention and control. The third section assessed participants' attitudes toward COVID-19, using a five-point Likert scale. For each of six statements, respondents were asked to state their level of agreement, from “strongly disagree,” “disagree,” “undecided,” “agree,” or “strongly agree.” The final section of the questionnaire assessed the respondents' practices. This section consisted of five questions related to practices and behavior, including (a) going to social events with large numbers of people, (b) going to crowded places, (c) avoiding cultural behaviors, such as shaking hands (d) practicing social distancing, (e) washing hands after sneezing, coughing, nose-blowing, and, recently, being in a public place.

### Independent Variables

For sociodemographic variables, gender was coded as one for men, and zero for women. The age variable was divided into categories: 18–29 (reference category), 30–39, 40–49, 50–59, and ≥60. Marital status was captured as binary, and a value of one was used for marriage and zero for otherwise. Education was categorized into high school or below (reference category), college/university degree, and postgraduate degree. Work status was broken down into government employee (reference category), non-government employee, retiree, self-employed, and unemployed. Monthly income (Saudi Riyal, SR 1 = USD 0.27) was divided into eight categories: < SR 3,000 (reference category); SR 3,000 to <5,000, SR 5,000 to <7,000, SR 7,000 to <10,000, SR 10,000 to <15,000, SR 15,000 to <20,000, SR 20,000 to <30,000, and SR 30,000 or more. We also controlled for the 13 administrative regions: Almadina Almonawra, Albaha, Aljouf/Quriat, Aseer/Bisha, Eastern Region, Haiel, Jazan, Najran, Northern Borders, Qaseem, Riyadh, Tabouk, and the Western Region.

### Dependent Variables

Respondents were asked to respond to knowledge items as either true or false, with an additional “don't know” option. Incorrect or uncertain (don't know) responses were given a score of zero, and correct answers were assigned a score of one. The total score for knowledge ranged from zero to 22, with high scores indicating better knowledge of COVID-19. Items were evaluated for internal reliability, using Cronbach's α. Cronbach's alpha coefficient was 0.70, indicating internal reliability (20).

In the section on attitudes, scores were calculated based on the respondents' answers to each attitudinal statement, 1 = strongly disagree, 2 = disagree, 3 = undecided, 4 = agree, and 5 = strongly agree. Scores were calculated by averaging respondents' answers to the six statements. Total scores ranged from six to 30, with high scores indicating positive attitudes. The Likert scales were assessed for internal reliability, using Cronbach's α. Cronbach's alpha coefficient was 0.81, indicating internal reliability. In the section on practices, respondents were asked to respond “yes” or “no” to the items. A score of one was given to answers that reflected good practice, and a score of zero was given for answers that reflected bad practice. The total score ranged from zero to five, with high scores indicating better practices.

### Analysis Methods

This study employed primarily univariate and multivariable regression data analyses. Univariate analysis was used to tabulate the frequency of social and demographic statistics. One-way analysis of variance (ANOVA) was used to assess differences in mean values for KAP scores. Because the scores were continuous, the overall mean differences were estimated using a Bartlett test (21, 22). A multivariable linear regression analysis was performed, to identify factors related to knowledge, attitudes, and practice. All analyses were conducted using STATA software (StataCorp LP, Texas, USA).

### Ethical Approval

All procedures performed in this study, involving human participants, complied with the institutional and/or national research committee ethical standards, and the 1964 Helsinki declaration and subsequent amendments or equivalent ethical standards. The study was designed and conducted in accordance with the ethical principles established by King Abdulaziz University. Therefore, ethical approval was obtained from the Biomedical Ethics Research Committee, Faculty of Medicine, King Abdulaziz University (Ref-180-20).

## Results

### Social and Demographic Characteristics

A total of 3,427 participants completed the questionnaire. After excluding 39 respondents who reported living outside the KSA, the final sample consisted of 3,388 participants. Table 1 shows the social and demographic characteristics of the study participants. As shown in Table 1, the mean COVID-19 knowledge score was 17.96 (SD = 2.24, range: 3–22), and the overall accuracy rate for the knowledge test was 81.64% (17.96/22 ^*^ 100). The mean attitude score for COVID-19 was 28.23 (SD = 2.76, range: 6–30), indicating positive attitudes. The mean score for practices for COVID-19 was 4.34 (SD = 0.87, range: 0–5), indicating good practices. Of the total sample, 1966 (58.03%) were women, and 1422 (41.97%) were men.

**Table 1:** Social and demographic characteristics of the study participants.

**Variable**	**Mean**	**SD**	**Min**	**Max**	***N***	**%**
Knowledge score	17.96	2.24	3	22		
Attitude score	28.23	2.76	6	30		
Practice score	4.34	0.87	0	5		
**Gender**						
Female					1,966	58.03
Male					1,422	41.97
**Age**						
18–29					1,016	29.99
30–39					940	27.74
40–49					692	20.43
50–59					472	13.93
≥ 60					268	7.91
**Marital status**						
Not married					1,239	36.57
Married					2,149	63.43
**Education**						
High school or below					539	15.91
College/University degree					1,904	56.20
Postgraduate degree					945	27.89
**Work status**						
Government employee					1,320	38.96
Non-government employee					546	16.12
Retiree					314	9.27
Self-employed					135	3.98
Unemployed					1,073	31.67
**Monthly income**						
< SR 3,000					846	24.97
SR 3,000 to <5,000					293	8.65
SR 5,000 to <7,000					258	7.62
SR 7,000 to <10,000					356	10.51
SR 10,000 to <15,000					584	17.24
SR 15,000 to <20,000					472	13.93
SR 20,000 to <30,000					333	9.83
≥ SR 30,000					246	7.26
**Region**						
Albaha					15	0.44
Aljouf/Quriat					10	0.30
Almadina Almonawra					147	4.34
Aseer/Bisha					149	4.40
Eastern Region					166	4.90
Haiel					17	0.50
Jazan					19	0.56
Najran					16	0.47
Northern Borders					4	0.12
Qaseem					38	1.12
Riyadh					535	15.79
Tabouk					15	0.44
Western Region					2257	66.62

The majority of the sample (57.73%) were between the ages of 18 and 39. Of the participants, 2,149 were married (63.43%) and 1,239 were unmarried (36.57%). More than half of the sample (56.20%) had a college or university degree. Respondents were grouped according to monthly income, with 846 (24.97%) in the < SR 3000 group, and 246 (7.26%) in the ≥ SR 30,000 group. In terms of work status, 1,073 (31.76%) were unemployed, and 314 (9.27%) were retired. Tables 2–4 show the responses to items related to KAP towards COVID-19.

**Table 2:** Responses to the questionnaire on COVID-19 knowledge.

**Statements**	***N*** **(%)**	
	**Correct answer**	**Incorrect answer**
SARS-CoV-2 spreads from person-to-person within close distance of each other (approx. six feet).	1,715 (50.62)	1,673 (49.38)
SARS-CoV-2 spread through respiratory droplets, which occur when infected people cough and sneeze.	3,210 (94.75)	178 (5.25)
SARS-CoV-2 can be contracted by touching a surface or object, on which the virus is attached, and then touching one's mouth, nose, or, perhaps, eyes.	3,323 (98.08)	65 (1.92)
Close contact or eating wild animals causes COVID-19.	2,121 (62.60)	1,267 (37.40)
People infected with SARS-CoV-2 cannot transmit the virus to others when a fever is not present.	2,885 (85.15)	503 (14.85)
The main clinical symptoms of COVID-19 are fever, fatigue, dry cough, myalgia and shortness of breath.	3,321 (98.02)	67 (1.98)
Unlike the common cold, congestion, runny nose, and sneezing are less common in people infected with SARS-CoV-2.	2,383 (70.34)	1,005 (29.66)
Antibiotics are an effective treatment for COVID-19.	2,194 (64.76)	1,194 (35.24)
Currently, there is no effective cure for COVID-19, but early symptomatic and supportive treatment can help most patients recover from the diseases.	3,260 (96.22)	128 (3.78)
Older adults and those with serious chronic illnesses, such as heart or lung disease and diabetes, are at increased risk of developing more serious complications from COVID-19.	3,227 (95.25)	161 (4.75)
Not all people with COVID-19 have severe cases. Only older adults with chronic illnesses tend to be more severe.	3,220 (95.04)	168 (4.96)
Pregnant women are more susceptible to infections than non-pregnant women.	1,685 (49.73)	1,703 (50.27)
Children do not appear to be at higher risk for COVID-19 than adults.	1,800 (53.13)	1,588 (46.87)
It is not necessary for children or young people to take precautionary measures to prevent SARS-CoV-2 transmission.	3,259 (96.19)	129 (3.81)
After being in a public place, after nose-blowing, coughing or sneezing, people must wash their hands with soap and water, or use hand sanitizer containing at least 60% alcohol, for at least 20 seconds.	3,094 (91.32)	294 (8.68)
People should avoid touching their eyes, nose, and mouth with unwashed hands.	3,353 (98.97)	35 (1.03)
Ordinary residents can wear general medical masks to prevent the SARS-CoV-2 infection.	1,483 (43.77)	1,905 (56.23)
People should only wear a mask if they are infected with the virus, or if they are caring for someone with suspected SARS-CoV-2 infection.	2,257 (66.62)	1,131 (33.38)
Healthy food and drinking water increase the body's immunity and resistance to COVID-19.	2,986 (88.13)	402 (11.87)
Isolation and treatment of people infected with the SARS-CoV-2, are effective ways to reduce the spread of virus.	3362 (99.23)	26 (0.77)
People in contact with someone infected with SARS-CoV-2 should be immediately quarantined, in an appropriate location, for a general observation period of 14 days.	3,353 (98.97)	35 (1.03)
To prevent transmission of SARS-CoV-2, people must avoid going to crowded places and avoid taking public transport.	3,353 (98.97)	35 (1.03)

**Table 3:** Responses to attitudinal statements regarding COVID-19.

**Statement**	***N*** **(%)**				
	**Strongly disagree**	**Disagree**	**Neutral**	**Agree**	**Strongly agree**
It is important to keep my distance from others, to avoid spreading SARS-CoV-2.	49 (1.45)	18 (0.53)	42 (1.24)	616 (18.18)	2,663 (78.60)
Washing hands is essential to protect myself from COVID-19.	37 (1.09)	5 (0.15)	8 (0.24)	367 (10.83)	2,971 (87.69)
To protect myself from COVID-19 exposure, I should stay home if I am sick, unless I am receiving medical care.	105 (3.10)	103 (3.04)	100 (2.92)	771 (22.76)	2,309 (68.15)
COVID-19 will eventually be successfully controlled.	29 (0.86)	15 (0.44)	147 (4.34)	778 (22.96)	2,419 (71.40)
Saudi Arabia's strict measures can help win the battle against COVID-19.	28 (0.83)	10 (0.30)	66 (1.95)	654 (19.30)	2,630 (77.63)
Compliance with the Ministry of Health precautions will prevent the spread of COVID-19.	25 (0.74)	6 (0.18)	21 (0.62)	485 (14.32)	2,851 (82.15)

**Table 4:** Practices related to COVID-19.

**Statement**	***N*** **(%)**	
	**Yes**	**No**
Have you recently been to a social event involving a large number of people?	169 (4.99)	3,219 (95.01)
Have you recently been to a crowded place?	205 (6.05)	3,183 (93.95)
Have you recently avoided cultural behaviors, such as shaking hands?	2,967 (87.57)	421 (12.63)
Have you been practicing social distancing?	2,867 (84.62)	521 (15.38)
Recently, have you frequently washed your hands with soap and water, for at least 40 seconds, especially after going to a public place, or after nose-blowing, coughing, or sneezing?	2,476 (73.08)	912 (26.92)

**Figure 1:**
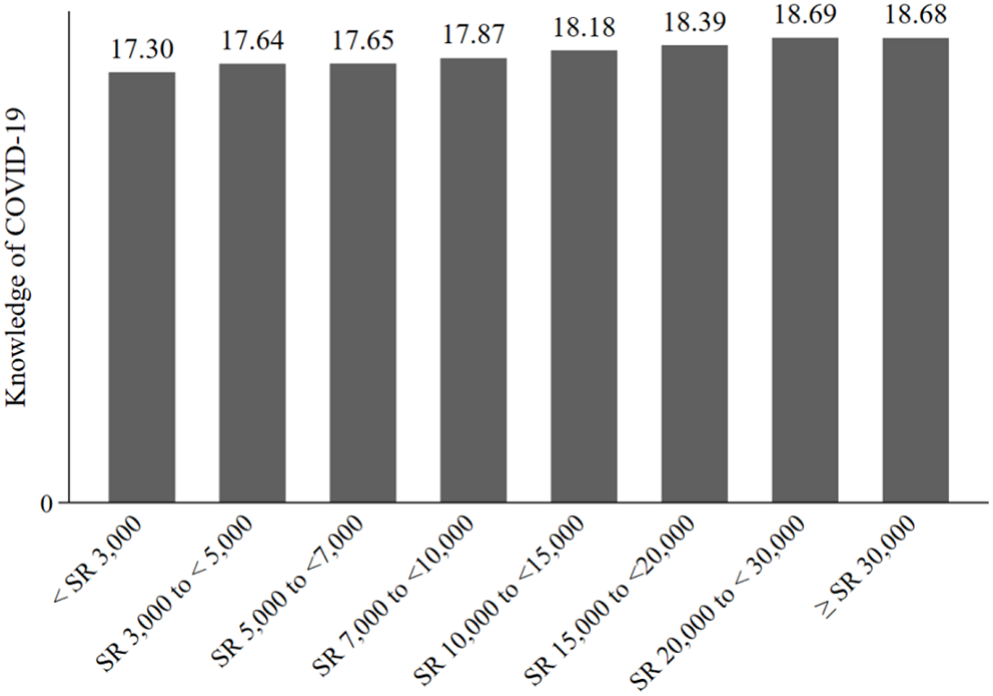
Knowledge of COVID-19, by income group.

**Figure 2:**
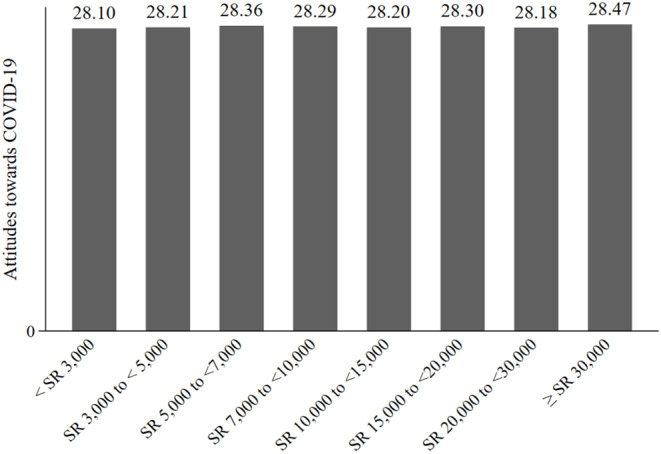
Attitude toward COVID-19, by income group.

**Figure 3:**
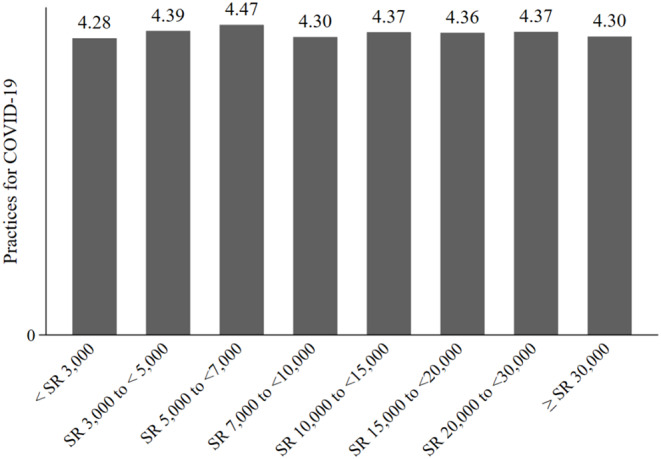
Practices for COVID-19, by income group.

We also assessed the level of KAP, across the various income groups. Figures 1–3 show the results. Figure 1 shows that the COVID-19 knowledge score increases with income. The lowest score was for respondents in the low-income category, < SR 3000, and the highest score was for respondents with an income of SR 20,000 to <30,000. For attitudes, Figure 2 shows that there were no discernible patterns across income groups. Furthermore, with regard to practices, there was little variation between income groups, as shown in Figure 3.

### Differences in Knowledge, Attitudes, and Practices Toward COVID-19

Looking at the univariate statistics for each variable of interest, we took another step to assess the difference in the scores for KAP. Table 5 shows the results.

**Table 5:** Comparison of social and demographic characteristics, and mean KAP score.

**Variable**	**N**	**%**	**Knowledge score**			**Attitude score**			**Practice score**		
			**Mean**	**SD**	***P***	**Mean**	**SD**	***P***	**Mean**	**SD**	***P***
**GENDER**											
Female	1,966	58.03	17.95	2.24	0.7241	28.35	2.32	<0.001	4.45	0.78	<0.001
Male	1,422	41.97	17.97	2.25		28.06	3.27		4.20	0.95	
**AGE**											
18–29	1016	29.99	17.17	2.44		28.22	2.30		4.26	0.90	
30–39	940	27.74	18.24	2.05		28.34	2.68		4.38	0.85	
40–49	692	20.43	18.14	2.13	<0.001	28.21	3.20	<0.001	4.37	0.89	0.019
50–59	472	13.93	18.52	2.08		28.10	3.05		4.42	0.79	
≥60	268	7.91	18.50	1.87		28.15	2.89		4.29	0.83	
**MARITAL STATUS**											
Not married	1,239	36.57	17.47	2.35	<0.001	28.26	2.39	<0.001	4.33	0.86	0.580
Married	2,149	63.43	18.24	2.13		28.21	2.96		4.35	0.87	
**EDUCATION**											
High school or below	539	15.91	17.22	2.64		28.01	2.81		4.26	0.88	
College/University degree	1,904	56.20	17.91	2.17	<0.001	28.26	2.72	0.38	4.34	0.88	0.028
Postgraduate degree	945	27.89	18.48	2.02		28.29	2.82		4.40	0.82	
**WORK STATUS**											
Government employee	1,320	38.96	18.24	2.14		28.28	3.10		4.34	0.88	
Non-government employee	546	16.12	17.91	2.28		28.29	2.25		4.37	0.86	
Retiree	314	9.27	18.50	1.83	<0.001	28.16	2.87	<0.001	4.31	0.84	0.677
Self-employed	135	3.98	18.15	2.02		27.96	3.30		4.51	0.83	
Unemployed	1,073	31.67	17.46	2.39		28.18	2.43		4.32	0.86	
**MONTHLY INCOME**											
< SR 3,000	846	24.97	17.30	2.40		28.10	2.55		4.28	0.89	
SR 3,000 to <5,000	293	8.65	17.64	2.33		28.21	2.56		4.39	0.83	
SR 5,000 to <7,000	258	7.62	17.65	2.24	<0.001	28.36	2.91	<0.001	4.47	0.81	0.04
SR 7,000 to <10,000	356	10.51	17.87	2.10		28.29	2.82		4.30	0.95	
SR 10,000 to <15,000	584	17.24	18.18	2.16		28.20	2.90		4.37	0.86	
SR 15,000 to <20,000	472	13.93	18.39	2.00		28.30	3.02		4.36	0.81	
SR 20,000 to <30,000	333	9.83	18.69	1.93		28.18	3.11		4.37	0.85	
≥ SR 30,000	246	7.26	18.68	2.09		28.47	2.00		4.30	0.87	
**REGION**											
Albaha	15	0.44	18.07	1.53		28.60	1.24		4.20	0.86	
Aljouf/Quriat	10	0.30	17.70	1.95		27.90	2.08		3.70	1.42	
Almadina Almonawra	147	4.34	18.30	2.19		27.84	3.69		4.14	0.98	
Aseer/Bisha	149	4.40	18.09	2.13		28.40	2.92		4.10	1.01	
Eastern Region	166	4.90	18.06	2.11		28.17	3.64		4.34	0.81	
Haiel	17	0.50	17.88	1.73	0.5309	27.71	2.76	<0.001	4.12	0.99	<0.001
Jazan	19	0.56	17.89	1.66		27.79	1.90		4.16	1.07	
Najran	16	0.47	17.81	1.64		27.38	6.01		4.25	1.13	
Northern Borders	4	0.12	18.50	1.91		21.50	10.54		5.00	0.00	
Qaseem	38	1.12	17.87	2.22		28.47	2.01		3.55	1.11	
Riyadh	535	15.79	18.12	2.28		28.13	2.92		4.28	0.87	
Tabouk	15	0.44	18.53	1.85		27.93	2.15		4.20	0.94	
Western region	2257	66.62	17.88	2.27		28.29	2.52		4.41	0.83	

As shown in Table 5, all scores for KAP, were statistically different at the 1% significance level, for all age and income groups. Although, across genders, there was a statistically significant difference in attitudes and practices scores, there was no difference in knowledge scores. Although assessments showed that knowledge scores were not statistically different between regions, attitudes and practices scores were significantly different at the 1% level.

### Econometric Results

Apart from the univariate and non-parametric analyses performed in previous sections, we also focused on regression analysis. Scores were logged for all variables, and interpreted using ordinary least squares (OLS). Increased scores imply increased knowledge, practices, and attitudes. The results are shown in Table 6.

**Table 6:** Regression results of KAP-related factors for COVID-19.

**Variable**	**Knowledge**	**Attitude**	**Practice**
	****β (*SE*)****	****β (*SE*)****	****β (*SE*)****
**GENDER**			
Male	−0.018^***^	−0.018^***^	−0.064^***^
	(0.006)	(0.006)	(0.010)
**AGE**			
30–39	0.047^***^	−0.004	0.039^***^
	(0.009)	(0.007)	(0.014)
40–49	0.041^***^	−0.014	0.033^**^
	(0.010)	(0.009)	(0.016)
50–59	0.057^***^	−0.021^**^	0.051^***^
	(0.011)	(0.009)	(0.018)
≥60	0.051^***^	−0.020	0.022
	(0.013)	(0.016)	(0.024)
**MARITAL STATUS**			
Married	0.009	−0.004	−0.002
	(0.006)	(0.005)	(0.011)
**EDUCATION**			
College/University degree	0.040^***^	0.000	0.008
	(0.009)	(0.007)	(0.012)
Postgraduate degree	0.050^***^	−0.005	0.012
	(0.010)	(0.010)	(0.015)
**WORK STATUS**			
Non-Government employee	0.010	0.005	0.014
	(0.008)	(0.009)	(0.014)
Retiree	0.013	0.008	−0.005
	(0.009)	(0.014)	(0.021)
Self-employed	0.010	−0.014	0.043^*^
	(0.011)	(0.017)	(0.024)
Unemployed	0.012	0.002	0.009
	(0.010)	(0.010)	(0.016)
**MONTHLY INCOME**			
SR 3,000 to <5,000	0.004	0.008	0.015
	(0.012)	(0.010)	(0.017)
SR 5,000 to <7,000	−0.000	0.011	0.032^*^
	(0.014)	(0.013)	(0.019)
SR 7,000 to <10,000	0.013	0.015	−0.001
	(0.013)	(0.012)	(0.019)
SR 10,000 to <15,000	0.021^*^	0.016	0.022
	(0.013)	(0.013)	(0.018)
SR 15,000 to <20,000	0.030^**^	0.020	0.018
	(0.013)	(0.013)	(0.018)
SR 20,000 to <30,000	0.043^***^	0.016	0.020
	(0.014)	(0.016)	(0.020)
≥ SR 30,000	0.041^**^	0.040^***^	−0.004
	(0.019)	(0.013)	(0.022)
**REGION**			
Albaha	0.025	0.033^**^	−0.008
	(0.022)	(0.014)	(0.067)
Aljouf/Quriat	−0.010	0.004	−0.195
	(0.035)	(0.023)	(0.154)
Almadina Almonawra	0.020	−0.015	−0.040
	(0.012)	(0.019)	(0.024)
Aseer/Bisha	0.009	0.013	−0.055^**^
	(0.012)	(0.016)	(0.028)
Eastern region	0.003	−0.007	0.012
	(0.012)	(0.018)	(0.021)
Haiel	−0.001	0.001	−0.031
	(0.021)	(0.025)	(0.065)
Jazan	0.024	−0.004	−0.051
	(0.022)	(0.017)	(0.080)
Najran	0.000	−0.059	−0.016
	(0.024)	(0.097)	(0.101)
Northern borders	0.015	−0.420	0.176^***^
	(0.047)	(0.327)	(0.018)
Qaseem	0.004	0.026^*^	−0.208^***^
	(0.025)	(0.015)	(0.060)
Tabouk	0.037	−0.001	−0.043
	(0.026)	(0.023)	(0.064)
Western region	−0.001	0.011	0.032^***^
	(0.007)	(0.007)	(0.012)
**Knowledge**		0.095^***^	0.167^***^
		(0.019)	(0.036)
_cons	2.786^***^	3.056^***^	0.926^***^
	(0.016)	(0.054)	(0.105)
***N***	3,388	3,388	3,380

Standard errors in parentheses,

*** p < 0.01,

** p < 0.05,

* *p < 0.1*.

Table 6 shows that, for knowledge of COVID-19, age groups 30–39, 40–49, 50–59 and ≥60, are more knowledgeable about COVID-19 than the reference group (18–29). All variables for age groups 30–39 (β = 0.047; *p* < 0.001), 40–49 (β = 0.041; *p* < 0.001), 50–59 (β = 0.057; *p* < 0.001) and ≥60 (β = 0.051; *p* < 0.001), are statistically significant at the 1% level. However, attitudes follow a different trend. Only the age group 50–59 (β = −0.021; *p* < 0.001) is significantly different from baseline. In practices for COVID-19, age groups 30–39 (β = 0.039; *p* < 0.001), 40–49 (β = 0.033; *p* < 0.05), and 50–59 (β = 0.051; *p* < 0.001), are associated with good practices.

Regarding gender, the results indicate that, compared to women, men have lower knowledge (β = −0.018; *p* < 0.001), lower positive attitudes (β = −0.018; *p* < 0.001), and few good practices for COVID-19 (β = −0.064; *p* < 0.001). No difference in KAP toward COVID-19 was observed by marital status. However, the relationships between income, region, education, and variables of interest, are heterogeneous, suggesting substantial differences.

We also examined the association between KAP scores. Because all were logs, the interpretation would be akin to the elasticity. Therefore, Table 6 shows that every increase of 1% in knowledge score is associated an increase in attitude and practices scores, of 0.095 and 0.16, respectively.

## Discussion

COVID-19 is an emerging infectious disease that poses a significant threat to public health. Given the serious threats imposed by COVID-19 and the absence of a COVID-19 vaccine, preventive measures play an essential role in reducing infection rates and controlling the spread of the disease. This indicates the necessity of public adherence to preventive and control measures, which is affected by their knowledge, attitudes, and practices (KAP). Thus, this study aimed to assess the KAP of the Saudi population, for the novel coronavirus disease 2019, COVID-19.

Our findings indicate that most study participants were knowledgeable about COVID-19. Study participants achieved a mean of 81.64% in the knowledge questionnaire. This finding is consistent with other studies that have shown satisfactory levels of knowledge, across the Saudi population, for epidemics, such as MERS (23, 24). In our study, the high rate of correct answers to knowledge-related questions among participants, was not surprising. This may be due to the characteristics of the sample, as 84% had a college or university degree, or above, and 70% were over 30 years old. It may also be due to the distribution of the questionnaire, amid the COVID-19 outbreak. In that time, people may have gained awareness and knowledge about the disease and its transmission, via television, news and media platforms, to protect themselves and their families. The positive association found between knowledge, and educational background and age, supports our claim.

Most of the participants in our study (98%) were aware of the clinical symptoms, and 96% knew that there is no clinically approved treatment for COVID-19 as of the date of this manuscript. Viral infections have been documented to be highly contagious among people in close proximity (19). However, approximately half of the respondents were unaware that SARS-CoV-2 could spread from person-to-person in close proximity. It was also evident that the current general population (44%) had little knowledge of when and whom wearing masks to prevent infection. According to the WHO and the CDC, faces mask should only be worn by those who are sick or caring for people suspected of having COVID-19 (9, 19). These findings highlight the need to continue to encourage and emphasize maintaining social distancing, as a means of preventing the spread of the virus.

It is important to note that there has been a great deal of efforts at all levels by the government, including public awareness campaigns. The Saudi Arabian Ministry of Health (MOH) has conducted an intensive awareness campaign, communicated via its website, television and various social media. The MOH has produced a guide to COVID-19, to provide residents with facts and precautionary messages in more than 10 languages. The MOH also works with the public and the media, especially via social media platforms. These early actions on engaging the public in prevention and control measures, as well as efforts to combat rumors and misinformation, have been greatly expanded (25). It is worth noting that the KSA is in the unique position of having dealt successfully with two outbreaks of viral origin, of related viruses (26–30). This unique experience has helped the government in taking prompt response and precautionary measures against COVID-19 to control its spread.

Significant predictors of participant knowledge in this study were age, gender, educational level, and income level. This finding is supported by other studies that have found that older, female, and more educated respondents are more knowledgeable about emerging communicable diseases (23, 31). We also found that high income earners are more knowledgeable about COVID-19. Education, age, and income have been documented to be highly relevant to knowledge (32). Our findings suggest that greater emphasis should be placed on mass media, to target low-income, low-educated, young people, and men to improve public knowledge on the COVID pandemic, through awareness-raising interventions.

Concerning attitudes, participants showed a positive and optimistic attitude toward COVID-19. Approximately 94% concur that the virus can be successfully controlled, and 97% are convinced that the Saudi government will control the pandemic. Positive attitudes and high confidence in the control of COVID-19 can be explained by the government's unprecedented actions and prompt response in taking stringent control and precautionary measures against COVID-19, to safeguard citizens and ensure their well-being. These measures include the lockdown, and the suspension of all domestic and international flights, prayer at mosques, schools and universities, and the national curfew imposed on citizens. This finding is consistent with a recent study conducted in China, where the majority of participants were convinced that the disease is curable and that their country will combat the disease (33). However, these results contrast with other findings that suggest people tend to express negative emotions, such as anxiety and panic, during a pandemic that could affect their attitude (34).

Nevertheless, our results show that the participants' high knowledge of COVID-19 translates into good and safe practices, during the COVID-19 pandemic, which suggests that the practices of Saudi residents are very cautious. Almost 95% of respondents refrained from attending social events, 94% avoided crowded places, and 88% avoided shaking hands. Respondents adopted good and safe practices, as a result of Saudi Arabia's health authorities providing education and outreach materials, to increase public understating of the disease, and influence behavioral change.

Finally, the study findings may be useful to inform policymakers and healthcare professionals, on further public health interventions, awareness-raising, policies, and health education programs. Men were significantly less likely to have knowledge, optimistic attitudes, and appropriate or safe practices toward COVID-19. These findings are consistent with other studies showing that, in response to SARS and MERS, men were significantly less likely take preventive and protective measures than women (24, 35, 36). Our finding suggests that targeted health education interventions should be directed to this particular vulnerable population at high risk of contracting COVID-19. For example, COVID-19 knowledge may increase significantly, if health education programs are specifically targeted at men. Health information can be sent to women (wives, sisters, mothers) who live with men, which may influence their practices, as suggested by a study in Hong Kong (35).

### Study Strengths and Limitations

To the researchers' knowledge, this is the first study to investigate KAP toward COVID-19, in the general population of the KSA. Data collection took place 2 weeks after the KSA confirmed its first COVID-19 case. Therefore, the prompt results may help health authorities to plan preventive strategies for future events. However, in interpreting the results of this study, some limitations should be considered. Data used in the analysis of this study were self-reported, which might suffer from reporting bias. Future research might employ administrative data to address this issue. Furthermore, there may be some endogenous variables: general attitude and expectation from the government, personal hygiene, for example. However, even if we were to undertake causality analysis, we could not be in a position to do proper econometric identification because the data we used could not have a valid instrument to eliminate the endogeneity.

Additionally, community-based national sampling surveys were not feasible during this particular period. As such, data were collected online, through self-reported questionnaires, depending on the authors' networks. Therefore, the majority of the respondents were in the western region, where most of the authors come from. Further research should cover the perceptions of all regions of the country. Finally, this study did not address causation. Therefore, the regression results should be interpreted as relevant, as some variables may be endogenous. That said, the implication is for the objective of future research to assess whether there is a relationship between COVID-19 knowledge and mortality, household consumption patterns, and the demand for unprescribed flue medication.

## Conclusions

This is the first study to investigate KAP for the COVID-19 outbreak, among the general population of Saudi Arabia. Our findings suggest that Saudi residents, especially women, have good knowledge, positive attitudes, and good practices toward COVID-19. Knowledge of the disease is considered the first stepping stone to any health education activity that is implemented. Knowing the causes and transmission sources of a disease, increases the likelihood that people will become more aware of the spread of communicable diseases, and of the preventive measures to slow transmission. The results of this study suggest that more emphasis should be placed on less educated, lower income, and men. The findings may help policymakers identify the target populations, for COVID-19 prevention and health education.

## Data Availability Statement

The datasets generated and/or analyzed during the current study are not publicly available due to privacy and confidentiality agreements as well as other restrictions, but are available from the corresponding author (Mohammed K. Al-Hanawi) on reasonable request.

## Author Contributions

All authors made substantial contributions to conception and design, acquisition of data, or analysis and interpretation of data, took part in drafting the article or revising it critically for important intellectual content, gave final approval of the version to be published, and agreed to be accountable for all aspects of the work.

## Conflict of Interest

The authors declare that the research was conducted in the absence of any commercial or financial relationships that could be construed as a potential conflict of interest.

## Abbreviations

COVID-19: Coronavirus disease 2019
KAP: Knowledge, attitude and practice
SARS: Severe acute respiratory syndrome
RNA: Ribonucleic acid
MERS: Middle East Respiratory Syndrome
WHO: World Health Organization
KSA: Kingdom of Saudi Arabia
CDC: Centres for Disease Control and Prevention
SR: Saudi Riyal
USD: United States Dollar
ANOVA: One-way analysis of variance
OLS: Ordinary least squares
MOH: Ministry of Health.
